# Selection of inter-row herbaceous covers in a sloping, organic, non-irrigated vineyard

**DOI:** 10.1371/journal.pone.0279759

**Published:** 2022-12-30

**Authors:** Cristina Pornaro, Franco Meggio, Fulvio Tonon, Luca Mazzon, Luigi Sartori, Antonio Berti, Stefano Macolino

**Affiliations:** 1 Department of Agronomy, Food, Natural Resources, Animals and Environment, University of Padova, Legnaro (PD), Italy; 2 Interdepartmental Centre for Research in Viticulture and Enology, University of Padova, Conegliano (TV), Italy; 3 Laboratorio Rocce e Ricerca Tonon, s.r.l., Trento, Italy; 4 Department of Land Environment Agriculture and Forestry, University of Padova, Legnaro (PD), Italy; California State University Fresno, UNITED STATES

## Abstract

Inter-row vegetation in vineyards is classified as a service crop as it provides many ecosystem services. The vegetation is often removed but maintaining them can mitigate the negative effects on the environment. However, the type of species or mixture choice can affect their success. A field trial was conducted in an organically-managed vineyard of Cabernet sauvignon *Vitis vinifera* L. cultivars in north-eastern Italy, in which three blends of grass species (*Shedonorus arundinaceus*, *Lolium perenne* and *Festuca rubra*) and two grass-legume mixtures were grown in the inter-rows and compared with resident vegetation and regularly tilled bare soil. Each vegetation type, including resident vegetation, was subjected to mulching and non-mulching treatments. The aim of this study was to evaluate the use of seeded species or mixtures in the inter-row spaces of the vineyard in north-Italy as an alternative management to resident vegetation and tilled soil. The experiment was conducted over two years to monitor the persistence of the sown vegetation and the influence of vegetation types on vine performance and grape composition, and on soil compaction and erosion. The relative abundances of each species, vegetation height, percentage green cover and normalised difference vegetation index (NDVI), vine shoot length, number of leaves per vine shoot, leaf area, bunch weight, vine NDVI, soil compaction and erosion, and depth and width of tractor tyre prints were measured. Over time, weed invasion altered the botanical composition of all vegetation types except for the *S*. *arundinaceus* blend, which remained stable throughout the study period. Our results showed that vine parameters were not affected by the type of vegetation in the inter-rows, nor were there differences between the grassed and bare soil inter-rows. Soil compaction and erosion, and tractor tyre prints were not directly affected by the type of vegetation cover, but they were affected by tillage in the plots with bare soil in the inter-rows or where it was used to prepare the soil for sowing. Soil compaction and erosion were related to the percentage vegetation cover. Mulching did not affect any of the parameters measured. Therefore, species selection plays a crucial role in inter-row vegetation management and in minimising environmental impacts. *S*. *arundinaceus* gave high protection against soil erosion due to its high persistence throughout the year and had the lowest growth rate thus requiring fewer cuttings.

## Introduction

Viticulture is one of the world’s most important agricultural sector, covering 7.4 million ha in 2018 [1] across a very broad range of latitudes, climates, and soil types. Although vineyards are among the most intensively managed agroecosystems, with numerous pesticide applications, soil tillage operations and high landscape simplification [2], they also offer the potential for a broad range of essential ecosystem services, primarily through the inter-row alleys [3]. Inter-row vegetation is classified as a service crop [4] as it provides many benefits to the primary crop and the environment, such as reducing soil erosion, increasing soil water availability, weed and pest control, and improving traffic tolerance, soil fertility and biodiversity [5–13]. Inter-row vegetation is often removed due to perceived competition with vines for water and nutrients [14]. However, the effect of vegetation cover on vine yield is still not clear. Although some studies found the expected reduction in grape yield [e.g. 15, 16], similar or even higher yields have also been observed in vineyards retaining inter-row vegetation [17, 18]. In semi-arid environments, the soil is usually kept bare, while in regions with a less dry climate, different soil management strategies aimed at improving soil fertility and ecosystem services have been adopted [15]. In a meta-analysis of 74 studies comparing the effects of extensive and intensive management (such as soil tillage and herbicide use) of inter-row vegetation covering 13 wine‐producing countries, Winter et al. [13] found no overall negative effect on grape quantity or quality.

Several studies have reported inter-row vegetation cover as a means of reducing runoff and soil erosion in vineyards, with effectiveness varying according to local environmental conditions [19–22]. In contrast, intensive soil tillage and herbicide application have been reported to trigger soil erosion [23]. Panagos et al. [24] estimated soil loss in Europe for the reference year 2010 with a modified version of the Revised Universal Soil Loss Equation model (RUSLE2015) and reported a mean soil loss rate in the European Union’s erosion-prone lands (agricultural, forested and semi-natural areas) of 2.46 t ha^−1^ yr^−1^, resulting in a total annual soil loss of about 970 Mt. Perennial crops, including vineyards, had the highest soil erosion rates among agricultural land uses (9.47 t ha^−1^ yr^−1^), accounting for 10% of total soil losses in the 28 European Union countries. Furthermore, Maetens et al. [25] showed that in the Mediterranean region runoff coefficients higher than 9% were related to vineyard land use. Soil erosion is likely related to soil compaction caused by tractor traffic, which also influences soil water infiltration and retention [26]. Repeated tractor traffic in inter-row alleys causes soil compaction on most of the vineyard area [27], which is exacerbated when operations are performed on wet soil [28]. Soil compaction has been widely shown to have several negative impacts on soil physical properties and plant growth. It increases the soil’s resistance to root penetration, reduces yields [29, 30] and negatively affects the physical fertility and organic carbon stock of the soil. Studies conducted on the steep sloping vineyards of the Alto Monferrato region in north-western Italy have shown the effectiveness of permanent grass cover between the rows in reducing runoff and soil erosion rates compared to tilled soil [19, 31].

Organic farming relies mainly on locally available resources and key issues in this approach are the sustaining of ecosystem services and conservation of local agrobiodiversity [32, 33]. Several studies have shown that organic farming systems are beneficial for the biodiversity of agroecosystems [34, 35] and generally increase biodiversity by 30% [36]. These benefits are provided by sustainable practices, for example, the sowing of native species or forb-rich seed mixtures [37]. The expansion of organic production has encouraged the sowing of vegetation in the inter-row alleys as an alternative to the frequent application of herbicides [14]. The botanical composition of inter-row vegetation affects agroecosystem functioning [9, 38] and the ability of the inter-row vegetation itself to provide beneficial ecosystem services [39, 40]. Species should be selected according to their adaptability to the specific environmental conditions and management systems to ensure plant persistence and stability of the botanical composition. Studies in the Mediterranean area suggest using annual legume species, in areas with an annual rainfall below 700 mm [41–43]. Where the annual rainfall is above 700 mm, perennial grasses are preferable [44]. In dry areas, species and cultivars with shallow roots are recommended [45, 46]. Oligospecific mixtures (4–5 species) have been found to give better species and ground cover persistence than monostands, and greater biological and morphological diversity, hence greater resilience against biotic and abiotic stresses [47, 48]. However, sowing only one species with well-known, controlled characteristics is sometimes preferable [47] as it simplifies cultivation practices, although inter-row vegetation often consists of spontaneous species that can be maintained with only periodic cuts during the growing seasons [14].

The mown biomass is usually chopped into small pieces and left as a thin layer, a practice known as mulching [49–51]. Whether or not removal of the mown biomass is beneficial depends on soil nutrient availability. Removing the mown material without proper nutrient compensation with fertilizers impoverishes nutrient-rich sites and encourages the establishment of species-rich grasslands [52–55] or degrades nutrient-poor sites and is detrimental to the establishment and persistence of species-rich grasslands [56, 57]. In the latter case, removal of the mown biomass is not recommended as it is costly [49, 58, 59]. Mulching encourages grassland-internal nutrient cycling consisting in the decomposition of the mulching material, associated release of nutrients and their subsequent uptake by plants and soil organisms [60]. The effect of mulching on plant composition is usually studied in meadows as a possible alternative to regular mowing, especially where this is not feasible for economic or technical reasons [59, 60]. Several studies [e.g. 59, 61] have reported that mulching (2–3 cuts per growing season) did not preserve grassland species richness any better than not mulching, but it increased long-term biomass production, and has therefore mainly been recommended as an alternative to conventional cutting management to conserve species-rich grasslands [49, 62]. It has been shown that in low-production grasslands, mulching can be applied without substantial loss of plant species richness and diversity [63, 64]. In a study analysing the effects of mulching once a year over the course of eleven years, Gaisler et al. [65] found no substantial changes in the soil and herbage nutrient concentrations compared with the uncut and non-mulching treatments. However, 8 years into the study they found changes in the sward structure with the uncut and mulched-once-a-year treatments consisting in an increase in the tall/short species ratio [65].

To the best of our knowledge, few studies have investigated the use of different plant species in the inter-rows of vineyards, and especially the long-term changes in botanical composition. To fill this knowledge gap, we carried out a field trial in an organically-managed vineyard of *Vitis vinifera* L. cultivar Cabernet Sauvignon in the wine-growing district of the Euganean hills (Padua, north-eastern Italy). In most of the vineyards planted in Southern and North-Central Italy, experiencing frequent drought in summer, mowed resident vegetation is preferred to any sown cover crop [66, 67]. On the contrary, in North-Eastern Italy, due to more favourable climate conditions, most of the planted vineyards, both organically- and conventionally-managed, already use cover crops or let the resident vegetation grow in the vineyard alleys. Three blends of grass species and two grass-legume mixtures were sown in the inter-rows and compared with resident vegetation and regularly tilled soil. Each vegetation type, including the resident vegetation, was subjected to mulching and non-mulching treatments. The experiment was conducted over two years to investigate variations in the botanical composition and sward cover of inter-row vegetation types, and the effects on vines and soil. The aim of this study was to evaluate the use of seeded species or mixtures in the inter-row spaces of vineyards in North-Italy as an alternative management to resident vegetation and tilled soil. Specifically, we addressed the following questions: (i) does the botanical composition of the inter-rows change over time? (ii) how does inter-row vegetation perform in terms of persistency? (iii) does inter-row vegetation influence vine performance or grape composition? (iv) are soil compaction and erosion affected by the type of inter-row vegetation? (v) does mulching influence the above-mentioned factors?

## Material and methods

### Experimental site and design

The experiment was conducted from October 2018 to October 2020 at Il Mottolo farm (Arquà Petrarca, province of Padua, north-eastern Italy, 45°15’09 N, 11°42’09 E, 35 m a.s.l.) in a sloping (15%), organically-managed, non-irrigated commercial vineyard of *Vitis vinifera* L. cv. Cabernet Sauvignon, established in 2004 (14 years old). The soil composition was 39.6% sand, 41.7% silt and 18.7% clay. The area has a humid subtropical climate with a mean air temperature of 13.5 °C and a mean annual rainfall of 963 mm. Monthly mean air temperatures and precipitation during the study period are reported in Table 1 [68].

**Table 1:** Monthly mean air temperatures and monthly precipitation during the study period, and long-term averages (1994–2020) at Arquà Petrarca, north-eastern Italy.

	Air temperature (°C)				Precipitation (mm)			
Month	2018	2019	2020	1994–2020	2018	2019	2020	1994–2020
January	5.8	2.8	4.7	3.3	28	17	18	49
February	3.2	7.5	7.9	4.9	70	56	10	64
March	6.6	10.6	9.2	8.9	163	6	69	68
April	15.9	12.8	14.6	12.8	43	206	12	95
May	19.0	13.9	17.9	17.4	84	224	41	96
June	22.4	24.8	21.0	21.7	91	33	102	80
July	24.7	24.8	24.2	23.9	90	106	76	70
August	24.9	24.7	24.4	23.6	142	38	192	71
September	20.6	19.5	20.2	18.8	114	94	38	88
October	15.6	15.3	13.2	13.8	121	109	133	98
November	9.9	9.7	9.2	8.5	96	273	15	111
December	3.9	6.2	5.2	4.0	17	101	193	74
Annual	14.4	14.4	14.3	13.5	1058	1263	899	963

Seven types of inter-row cover were compared: three blends of cool-season grasses (S1, S2, and S3), two cool-season grass-legume mixtures (M1 and M2), inter-row soil covered by resident species (RV), and tilled soil (BS). The grass seed blends composed of three turf-type varieties of *Shedonorus arundinaceus* (S1), *Lolium perenne* (S2) and *Festuca rubra* (S3). The two grass-legume mixtures were composed of four or five species of selected low-growing grasses and *Trifolium repens* (M1 and M2). The varieties and sowing rates of each blend and mixture are reported in Table 2. All the blends and mixtures were sown in September 2018 after grape harvest, soil preparation, and fertilization. The blends were sown at 20 (S1), 10 (S2) and 15 kg ha^-1^ (S3), and the two mixtures at 10 kg ha^-1^ according to the local practice. Each vegetation type was cut three times per growing season (May, June, and September) using a rotary motor machine set at a height of 6 cm, with mulching (M) and without (NM). At each cut, the bare soil was tilled to keep the soil free of any vegetation.

**Table 2:** Species and cultivars of the three grass blends and two grass-legume mixtures sown in the vineyard inter-rows at Arquà Petrarca, north-eastern Italy.

Blend (S)/Mixture (M)	Species	Cultivar
S1	*Schedonorus arundinaceus*	Rhambler SRP
		Olympic Gold
		Lexington
S2	*Lolium perenne*	Ecologic
		Presidian
		New Orleans
S3	*Festuca rubra*	Maxima
		Reverent
		Kent
M1	*Lolium perenne* (45%)†	Stefani
	*Festuca rubra* (40%)	Reverent
	*Poa pratensis* (8%)	Balin
	*Trifolium repens* (7%)	G.Huia
M2	*Festuca rubra* (50%)	Gondolin
	*Poa pratensis* (30%)	Balin
	*Lolium perenne* (10%)	Option
	*Festuca ovina var*. *duriuscula* (5%)	Ridu
	*Trifolium repens* (5%)	Winterwhite

^†^Seed percentage by weight of each species in the mixture.

**Fig 1:**
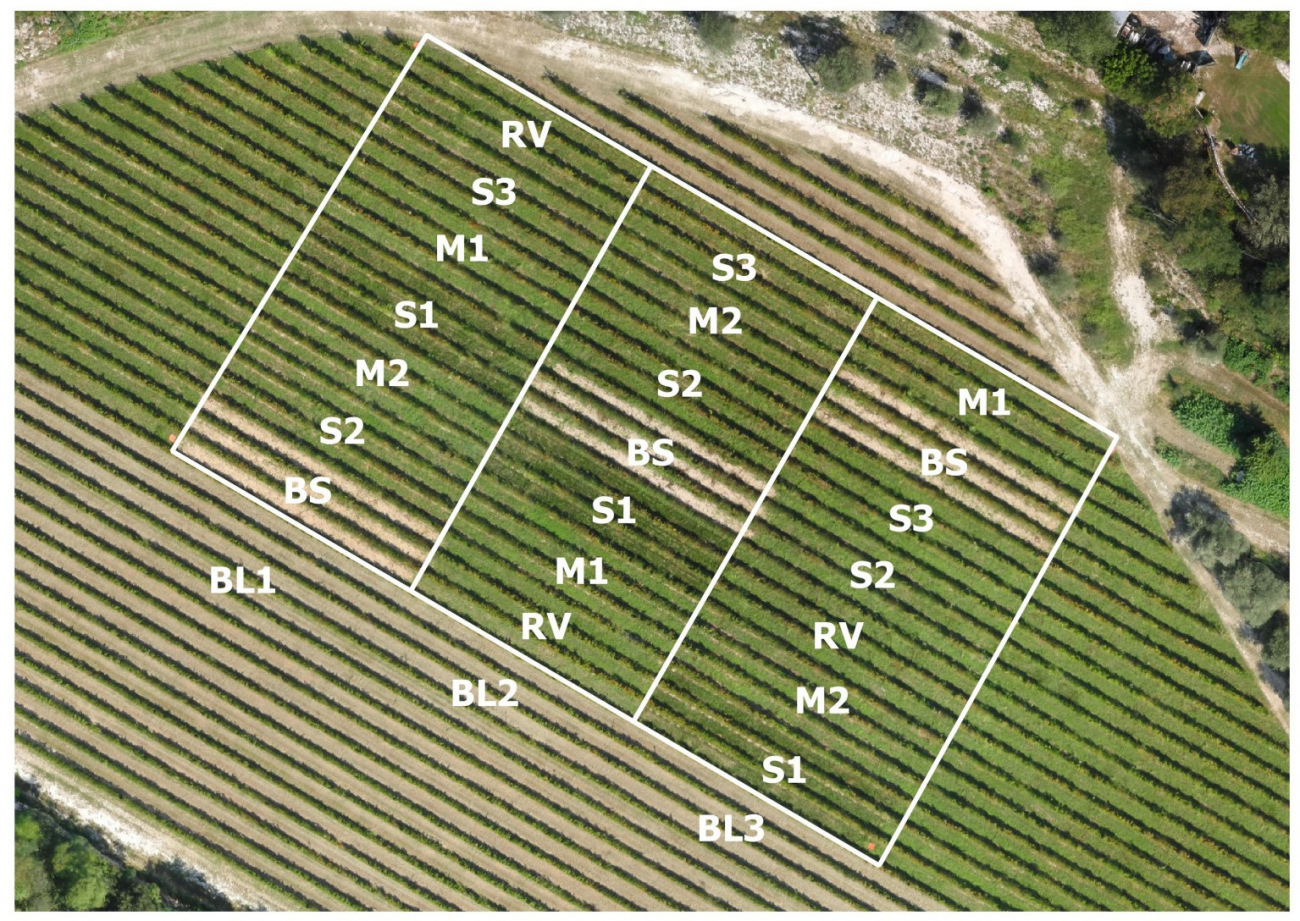
Plot arrangement for a field trial in a vineyard in Arquà Petrarca (Padua, north-eastern Italy, N 45°15’09, E 11°42’09, 35 m a.s.l.) (image acquired by Tonon F. on September 2020). S1, S2, and S3 = grass blends, M1 and M2 = grass-legume mixtures, BS = bare soil, RV = resident vegetation, BL1-BL3 = replications.

The experimental design was a strip-plot with three replications, with vegetation type as the whole plot and mulching as the strip plot (Fig 1). A given vegetation plot consisted of 3 adjacent inter-rows (1.30 m x 24 m each), and mulch was applied in a 12 m-wide strip across all vegetation types in each replication. The central inter-row plot was the test area.

The plots were not weeded before sowing or at any time during the experiment. A humidified pelleted manure (4 N– 4 P_2_O5−4 K_2_O) was applied at a rate of 70 kg ha^-1^ of N in March 2019 and 2020 prior to sowing the plots. Vine pest and disease control and other cultivation practices, including vine canopy management, were done according to normal practices during the growing season. Most of the farm operations in the vineyard were carried out using tracked or tyred tractors carrying or towing implements; these passed along each inter-row three times during the growing season in 2019 (May, June, and September), and twice in 2020 (May and September) to cut the inter-row vegetation, nine times from March to September for vine pest and disease control, and once at the beginning of October for grape harvest. The tractor model was New Holland TD4030F with an engine power of 57 kW, front tyres Goodyear DT812, 280/70 R16, rear tyres Goodyear DT812, 360/70 R24, for a total mass of 2.67 t.

### Plant species and botanical measurements

Botanical surveys of each sub-plot were conducted in June and August of both experimental years (2019 and 2020) using the vertical point quadrat method [69]. Two 10-m linear transects were placed on the two diagonals of the sub-plot, and the plant species touching a vertical steel needle inserted at 50-cm intervals along each transect were identified and recorded. Species nomenclature followed Pignatti [70]. In each survey, species relative abundances were calculated and used to determine the proportions of the different species according to Daget and Poissonet’s [69] equation. The data obtained from the botanical surveys were used to build species matrices for the analysis. In 2020, additional information on plant growth was gathered, which consisted of six measurements of vegetation height taken randomly in each sub-plot before each cut using a rising plate meter (mod. EC10; Grasstec Ltd, United Kingdom). One week after each cut, the percentage green vegetation cover was measured using digital image analysis [71]. Two digital images per sub-plot were taken with a Canon Powershot G12 installed on a light box, with a 1/400 s shutter speed, an F4.0 aperture, and a 32 mm focal length. Images were analysed using the ‘Turf Analysis’ macro [72] with the Sigma Scan Pro software (v. 5.0; SPSS Inc., Chicago, IL, USA) with a hue range from 57 to 107 and a saturation range from 0 to 100, to estimate the percentage green cover on the images. One week after each mowing, the normalised difference vegetation index (NDVI) was measured using a handheld crop sensor (GreenSeeker; Trimble Navigation Unlimited, Sunnyvale, CA, USA).

### Viticultural parameter assessment

At both the 2019 and 2020 harvest periods (2^nd^ October 2019 and 28^th^ September 2020), the following measurements were taken for each treatment: mean shoot length (sl), mean number of leaves per shoot (nl), and mean leaf area, destructively determined from 15 randomly selected vines in each sub-plot. All the leaves in each sample were removed and counted, and the specific surface area was measured using a Licor LI-3100c area meter (Li-Cor, Inc., Lincoln, NE, USA). The mean total leaf area of the shoots in each sub-plot was determined, and the leaf area index (LAI) was calculated from the mean of 12 shoots per vine and taking planting distance into account. The mean bunch weight (bw) per sub-plot was determined from 15 randomly selected bunches. After picking, the bunches were immediately brought to the laboratory and berry sugar content (°Brix) was measured in three replicates per treatment using a refractometer on 6 berries randomly selected from the cluster samples, 3 from the basal part and 3 from the head of each cluster.

**Fig 2:**
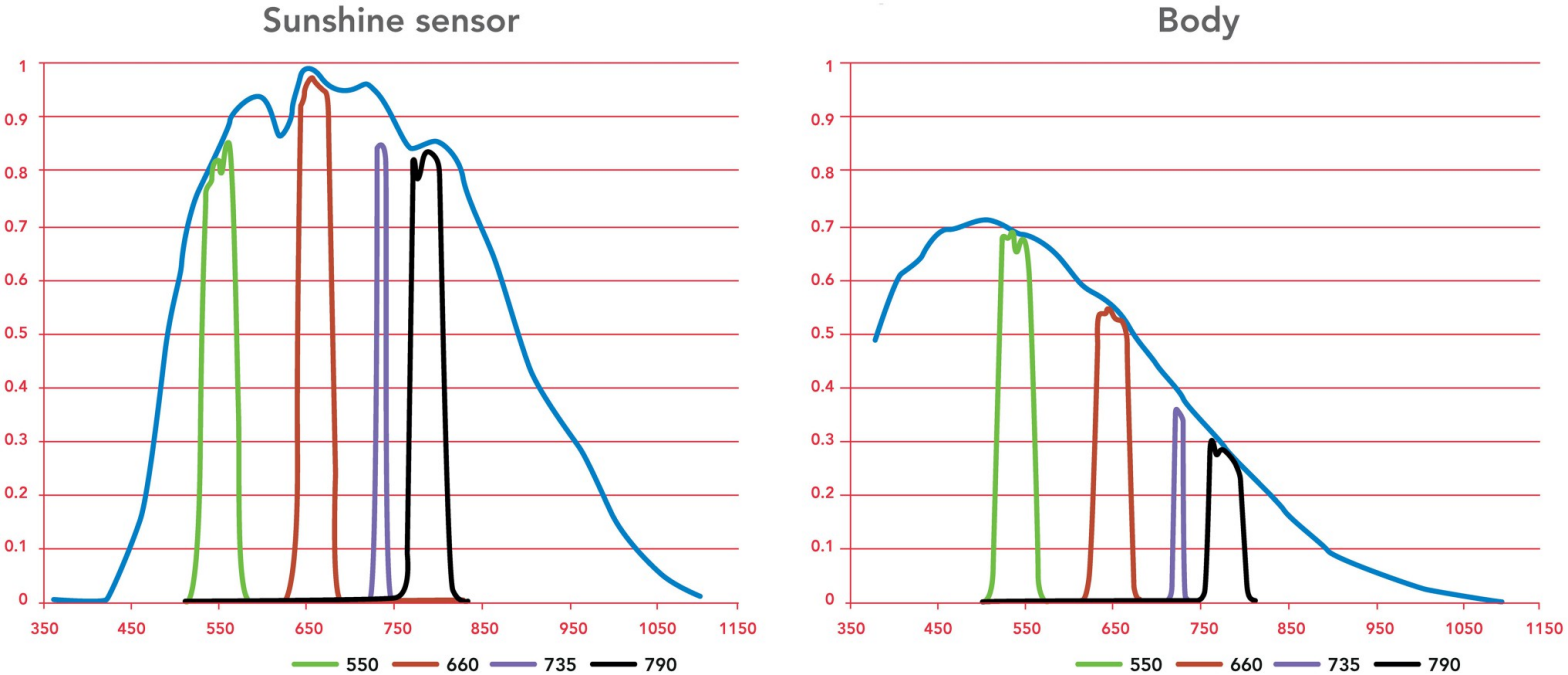
Sequoia sensitivities against wavelengths (nm) for the camera body and for the sunshine sensor: Green (550 nm), red (660 nm), red edge (735 nm), NIR (790 nm).

Aerial surveys were conducted on 9 September 2019, 8 August 2020 and 13 September 2020 to collect multispectral data of the canopy. For each survey, we used a SenseFly Ebee X fixed-wing unmanned aerial system (UAS) to capture RGB images with a SODA camera, and multispectral images with a Sequoia camera. Fig 2 shows the Sequoia sensitivities for the 4-channel camera body and for the sunshine sensor, which is used to modify the image data to take into account the solar energy hitting the leaves when a multispectral image is taken (this is similar to aperture adjustment in RGB photos). Before each flight, a set of calibration pictures was taken to set the correct reflectance on each of the four channels during the post-processing phase (this is similar to white balance calibration in RGB photos). All images were accurately georeferenced (to an accuracy of about 2 cm on the horizontal plane) with a PPK algorithm, and 1 s corrections logged at a local fixed GNSS station. This horizontal accuracy allows for a perfect overlay between the RGB and multispectral data from the same flight or from different flights; without such accuracy, horizontal shifts of at least 1 m would impede data interpretation, and correct attribution of, e.g., NDVI values to sub-plots. Orthophotos for the RGB spectrum and the NDVI indices were successfully produced for each flight.

In calculating the average NDVI over a sub-plot, it must be borne in mind that the NDVI is a ratio index, which is not equivalent to the proportion of vegetation (and which can have negative values). Therefore, for each sub-plot, first the average NIR and average RED were calculated over the sub-plot, and these values were then used to calculate the average NDVI over the sub-plot [73, 74].

### Soil compaction and erosion assessment

Soil compaction was evaluated in October of both 2019 and 2020 by measuring the maximum soil strength (N cm^-2^) at a depth of 0–10 cm five times in each sub-plot using a hand penetrometer (Eijkelkamp Agrisearch Equipment, Giesbeek, The Netherlands) with a cone size of 19 mm. Soil moisture content was also measured close to the site of each penetrometer measurement using a time domain reflectometer (FieldScout TDR 300; Spectrum Technologies Inc., Plainfield, IL, USA). In September of each year, before harvest, the depth and width of the tractor tyre prints were measured at three randomly selected points along the path of the tractor in each sub-plot. Soil erosion was measured by means of rainfall simulation in July on S1, RV and BS in 2019, and on S1, S2, RV and BS in 2020. To determine sediment detachment, heavy rainstorms (10 mm/min) were mimicked on a soil area of 0.24 m^2^ using a rainfall simulator consisting of a micro-perforated plastic tub (40 x 60 x 15 cm) placed horizontally at 1.60 m from the soil surface and containing 10 L of tap water. The eroded sediments were collected in tanks using a 25 x 60 cm metal sheet as a funnel placed at the base of the sampled areas. Soil erosion samples were oven dried at 105 °C to a constant weight to determine the sediment mass [75], and subsequently analysed in the laboratory to determine particle size distribution and total phosphorus (P) content. Particle-size distribution of sediments was analysed by laser diffraction methods, as described by Bittelli et al. [76]. Total P was estimated using method 3051A [77].

The various measurements and when they were taken are summarised in Table 3.

**Table 3:** List of measurements and month and year they were taken.

Measurement	2019						2020				
	April	June	July	Aug.	Sept.	Oct.	June	July	Aug.	Sept.	Oct.
Botanical survey		x^1^		x			x		x		
Vegetation height								x		x	
Percentage green cover	x		x		x			x		x	
Inter-row NDVI	x		x		x			x		x	
Mean shoot length (sl)						x					x
Mean no. of leaves/shoot (nl)						x					x
Leaf Area Index (LAI)						x					x
Mean bunch weight (bw)						x					x
Berry sugar content (bx)						x					x
Canopy NDVI				x					x		
Soil compaction						x					x
Tractor tyre print depth			x					x			
Tractor tyre print width			x					x			
Total runoff sediments					S2, BS, RV^2^					S1, S2, BS, RV	
Particle-size distribution of runoff sediments					S2, BS, RV					S1, S2, BS, RV	
Total P on runoff sediments					S2, BS, RV					S1, S2, BS, RV	

^1^ Measurements were collected from all sub-plots.

^2^ Measurements were collected from the specified sub-plots (S1 = *Shedondorus arundinaceus* blend; S2 = *Lolium perenne* blend; BS = bare soil; RV = resident vegetation)

### Data analyses

Vegetation height, percentage green cover and inter-row cover NDVI were used to parameterise a linear mixed model, where ’Sampling date’, ’Type of cover’, ’Mulching treatment’, and their interactions were included as fixed effects, while blocks within sampling date, and main plots within blocks were included as random effects to account for the clustering of observations and ensure the independence of the model residuals. NDVI, soil compaction, tractor tyre print depth, and tractor tyre print width in the vine rows were used to parameterise a linear mixed model, where ’Type of cover’, ’Mulching treatment’, ’Year’, and their interactions were included as fixed effects, while main plots within blocks was included as a random effect. Total runoff sediments in 2019, and in 2020, total P in runoff sediments in 2019, and in 2020 were used to parametrize a linear mixed model, where ’Type of cover’, ’Treatment’, and their interaction were included as fixed effects, and the main plots within blocks as random effects. Normality and homoscedasticity of the residuals were checked by graphical analyses. Fisher’s protected LSD test at a 0.05 level of probability was used to identify significant differences between means for significant variables.

We performed constrained correspondence analyses (CCA) to investigate the effects of types of inter-row cover and mulching (mowing with and without mulching) on the plant community composition, and a principal component analysis (PCA) on the particle-size distribution of runoff sediments. The general structure of the interdependences between treatment, physiological response and plant growth was explored with a correlation-based principal component analysis (PCA) on viticultural variables measured at harvest and averaged across seasons. Permutation tests were carried out to evaluate the significances of the explanatory variables in the CCA and the PCA.

Data were analysed with R version 4.0.2 [78], and additional packages: *vegan* for multifactorial analysis, *nlme* for fitting mixed models with repeated measures, and *multcomp* for post-hoc comparisons.

## Results

### Inter-row vegetation

**Fig 3:**
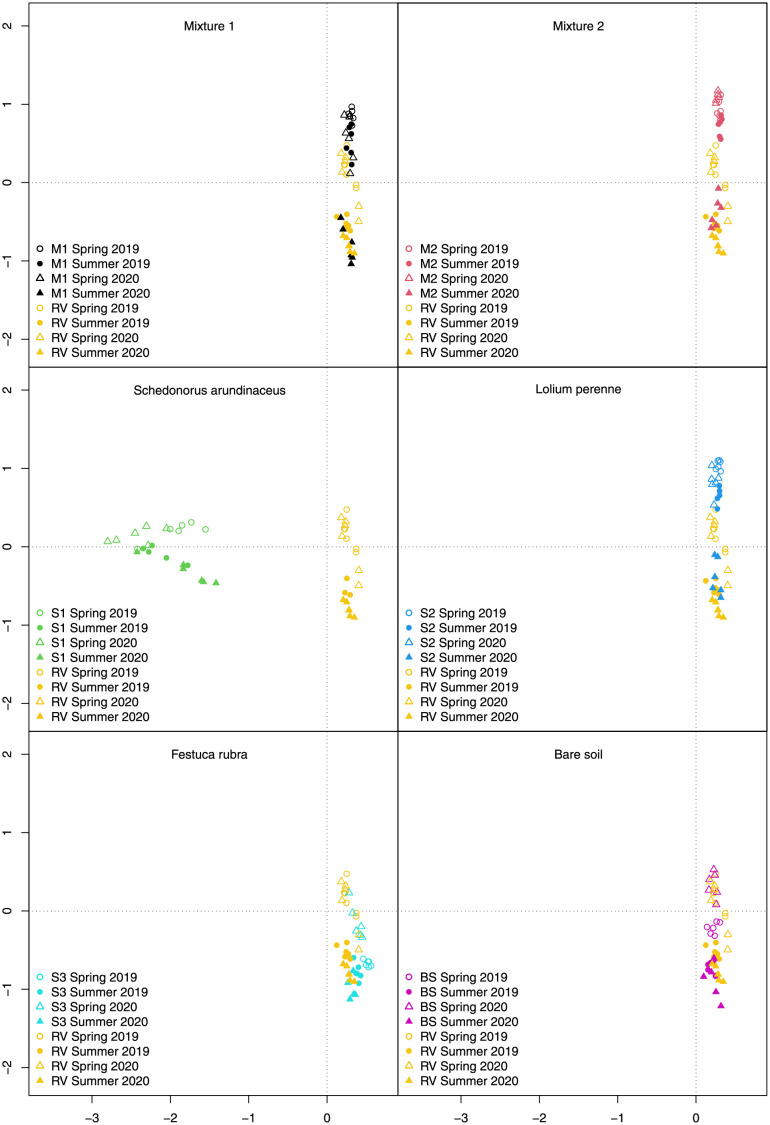
Results of canonical correspondence analysis of vascular plant species. Outlined symbols indicate surveys conducted in spring, solid symbols surveys conducted in summer (circles 2019, triangles, 2020). M1 = grass-legume mixture 1, M2 = grass-legume mixture 2, S1 = *Schedonorus arundinaceus* blend, S2 = *Lolium perenne* blend, S3 = *Festuca rubra* blend, RV = resident vegetation, BS = bare soil.

Inter-row botanical composition was significantly affected by vegetation type, but not by mulching (Table 4). The CCA analysis showed that the vegetation in the plots where *S*. *arundinaceus* was sown was clearly different from all the other plots in both 2019 and 2020 (Fig 3). The mixtures and S2 plots had similar compositions in spring and summer 2019, and in spring 2020 (Fig 3). However, the botanical composition of M1, M2, and S2 differed from RV in the spring after sowing, but there was a shift towards RV composition, especially with M1 in summer 2019 and spring 2020, and complete overlap in summer 2020. The *F*. *rubra* plots (S3) had a different botanical composition than the other sown vegetation types in 2019 (Fig 3) and shifted further towards resident vegetation in 2020. Furthermore, the botanical composition of RV overlapped with that of the bare soil (BS; Fig 3).

**Table 4:** Statistical significances based on a permutation test of the effects of type of inter-row vegetation, mulching and year on inter-row botanical composition, vine parameters and runoff sediment particle-size distribution in 2019 and 2020.

	Type of cover	Mulching	Year
Botanical composition	0.001	ns^†^	0.003
Vine parameters	ns	ns	0.003
Particle-size distribution, 2019	0.038	ns	-
Particle-size distribution, 2020	0.005	ns	-

^†^ns: Not significant at the 0.05 probability level.

**Fig 4:**
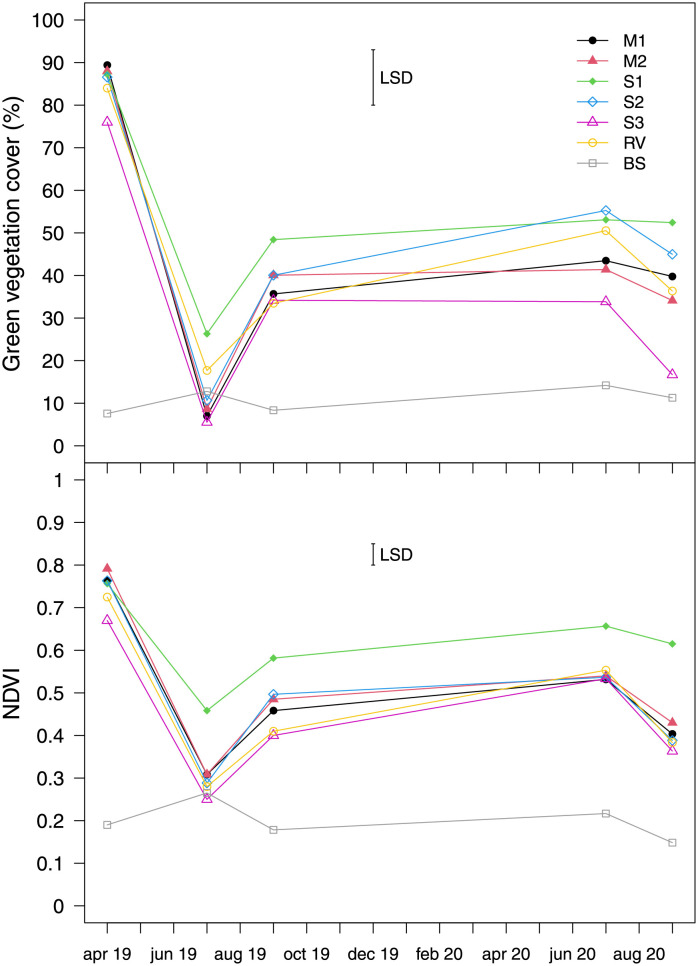
Effects of sampling date on the percentage green cover and NDVI of inter-row vegetation types (M1 = grass-legume mixture 1, M2 = grass-legume mixture 2, S1 = *Schedonorus arundinaceus* blend, S2 = *Lolium perenne* blend, S3 = *Festuca rubra* blend, RV = resident vegetation) and bare soil (BS). The vertical bars represent least significant differences (LSD) at a 0.05 probability level.

Percentage green cover and NDVI were significantly affected by the interaction between type of inter-row cover and sampling date (Table 5). As expected, BS had the lowest percentage cover and NDVI throughout the whole study period (Fig 4), except in July 2019 when the only type of vegetation with higher values than BS was S1. The highest values for percentage green cover were observed in spring 2019, immediately after sowing, with only S3 exhibiting a difference within type of vegetation. There was a rapid decrease in green cover in the summer, followed by a recovery in September and stabilisation of the values at a level that was also maintained in summer 2020. The only type of vegetation with reduced green cover from July to September 2020 was S3. In July 2019, S1 reached higher a percentage than the other vegetation types with the exception of RV, while in September 2019, S1 had higher values than M1, S3 and RV. In July and September 2020, S3 had lower values than S1 and S2. The trend in NDVI reflected that in vegetation cover (Fig 4), with some important differences: S1 had lower values than the other types of sown vegetation in April 2019, but higher values than all the other vegetation types in July 2019 and in July and September 2020.

**Table 5:** Results of the analysis of variance on the effects of type of inter-row vegetation (Ty), mulching treatment (M), and sampling date (Da), and their interactions on a) vegetation height, percentage green cover, and inter-row NDVI; b) vineyard NDVI, soil compaction, tractor tyre print depth, and tractor tyre print width; and c) total runoff sediments in 2019 and in 2020, and total P in runoff sediments in 2019 and 2020.

	Ty	M	Da	Ty x M	Ty x Da	M x Da	Ty x M x Da
Green cover	<0.001	ns^†^	<0.001	ns	<0.001	ns	ns
Inter-row NDVI	<0.001	ns	<0.001	ns	<0.001	ns	ns
Vegetation height	<0.001	ns	ns	ns	ns	ns	ns
Vine row NDVI	<0.001	ns	0.001	ns	ns	ns	ns
Soil compaction	<0.001	ns	0.007	ns	ns	ns	ns
Tractor tyre print depth	0.002	ns	ns	ns	ns	ns	ns
Tractor tyre print width	ns	ns	ns	ns	ns	ns	ns
Total runoff sediments, 2019	0.031	ns	-	ns	-	-	-
Total runoff sediments, 2020	<0.001	ns	-	ns	-	-	-
Total P on runoff sediments, 2019	0.013	ns	-	ns	-	-	-
Total P on runoff sediments, 2020	0.002	ns	-	ns	-	-	-

^†^ns: Not significant at the 0.05 probability level.

**Fig 5:**
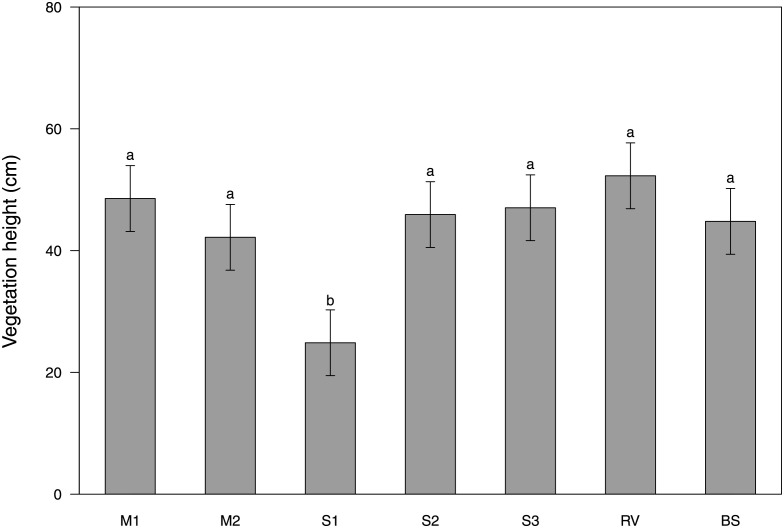
Effects of type of inter-row vegetation (M1 = grass-legume mixture 1, M2 = grass-legume mixture 2, S1 = *Schenodorus aundinaceus* blend, S2 = *Lolium perenne* blend, S3 = *Festuca rubra* blend, RV = resident vegetation) and bare soil (BS) on vegetation height. Values with the same letter are not significantly different (LSD test at a 0.05 probability level).

Vegetation height was affected by the type of inter-row vegetation (Table 5), with S1 being significantly shorter than all the other types of vegetation tested (Fig 5).

### Vine data

**Fig 6:**
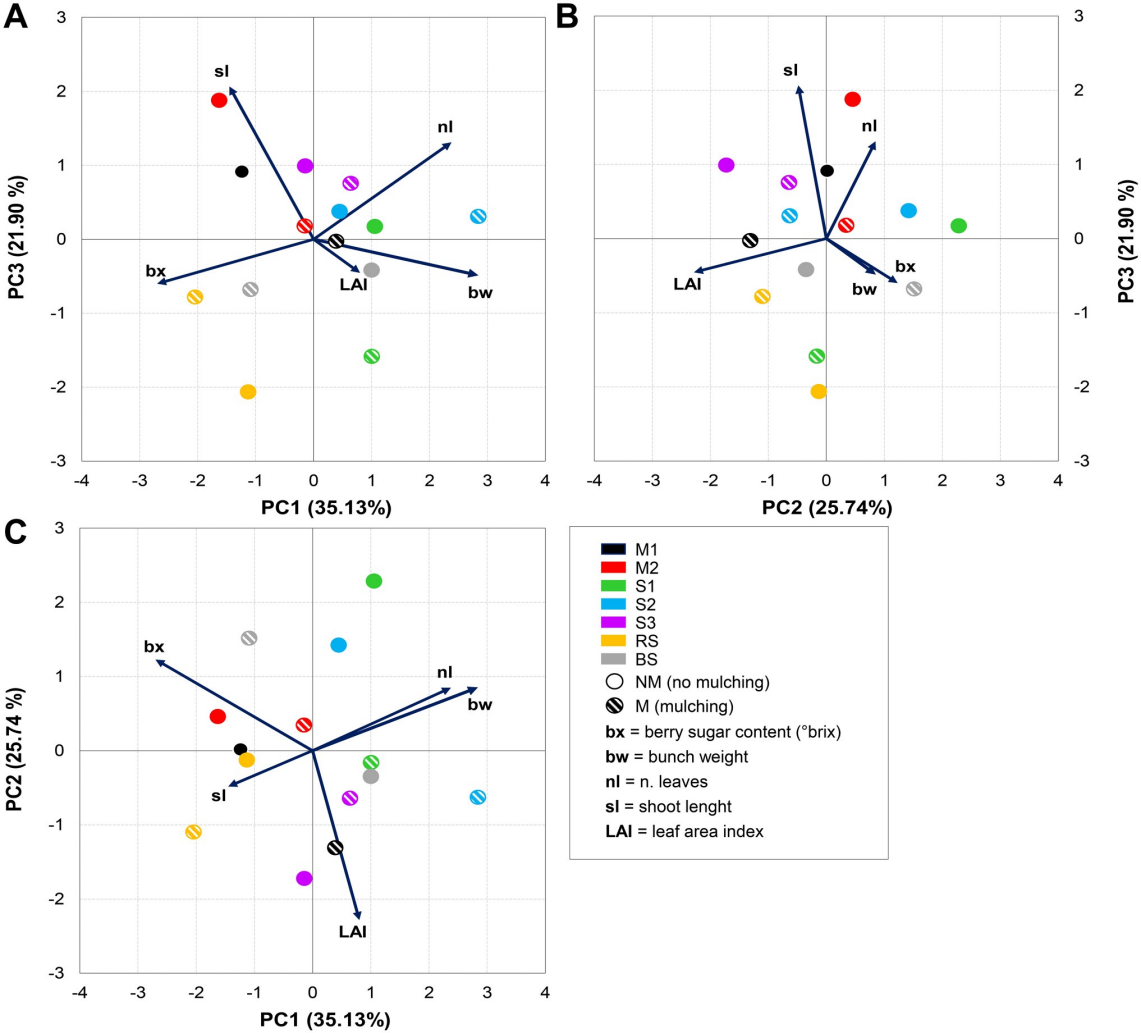
Site score plots of the selected vine parameters on the three principal components (PC1, PC2, PC3). Plotted points refer to the treatments tested (circles) and the effects of mulching (lined and solid). Vectors indicate the direction and strength of each variable to the overall distribution.

A significant effect of year was found for the vine parameters (Table 4), but not for type of inter-row cover and mulching treatment. A PCA was performed to establish the general structure of the interdependences between the changes in the inter-row vegetation type (M1, M2, S1, S2, S3, RV, BS), mulching treatment (M or NM), and fluctuations in the selected viticultural parameters (nl, sl, LAI, bw, bx) averaged over the two seasons. Three components were extracted from the PCA explaining more than 80% of the total variance (Fig 6). The first component, which accounted for 35.13% of the variance, was highly positively correlated (factor loadings ≥ 0.5) with bunch weight (bw), and negatively correlated with berry sugar content (bx). The second component explained 25.74% of the variance and was highly negatively correlated with the leaf area index (factor loading ≥ -0.78), while the third component explained 21.9% of the variance and was highly positively correlated with shoot length (factor loadings ≥ 0.79). Leaf number was positively correlated with PC1 and PC3 (factor loadings ≥ -0.5).

It is worth noting that, for each treatment, the effect of mulching can be illustrated by considering the shift from the non-mulching (NM) to the mulching (M) treatments. In particular, the effect of mulching on cool-season, grass-legume mixtures M1 and M2 shifted towards the LAI, nl and bw eigenvectors and away from the sl and bx eigenvectors, revealing a positive correlation with leaf growth in terms of size, which sustained carbohydrate production and in turn increased bunch weight (bw). In contrast, a negative correlation was observed on shoot length growth and berry sugar content.

Regarding the three blends of cool-season grasses (S1, S2, and S3), while mulching tended to affect LAI with S1, with S2 and S3 the effect was rather on nl and bw. In both cases, mulching seemed to reduce shoot length and berry sugar content. The control treatments—bare soil (BS) and resident vegetation (RV)—showed the opposite behaviour to the cover-crop species treatments. Mulching seemed to reduce the grass-vine competition, with the RV treatment promoting shoot length and BS treatment berry sugar content (Fig 6).

**Fig 7:**
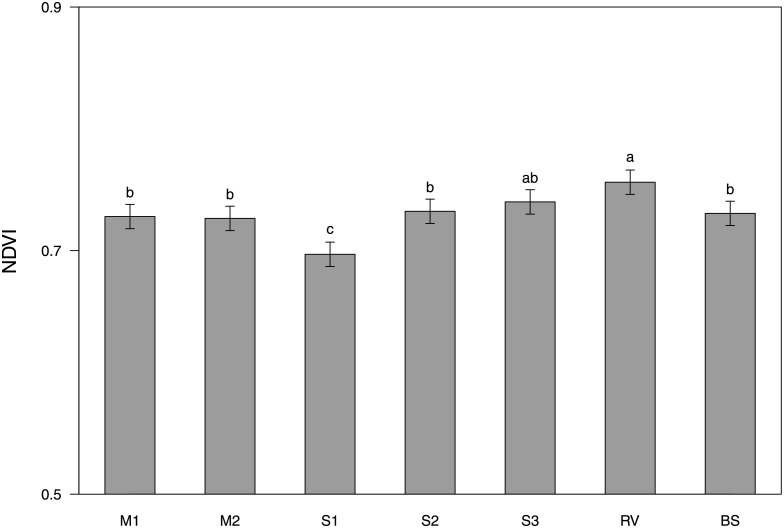
Effects of type of inter-row vegetation (M1 = grass-legume mixture 1, M2 = grass-legume mixture 2, S1 = *Schenodorus aundinaceus* blend, S2 = *Lolium perenne* blend, S3 = *Festuca rubra* blend, RV = resident vegetation) and bare soil (BS) on the NDVI values of the vine rows. Values with the same letter are not significantly different (LSD test at a 0.05 probability level).

The NDVI values for the vine rows were affected by type of inter-row vegetation (Table 5), with S1 having significantly lower values than all the others type of vegetation tested (Fig 7).

### Soil observation

Soil compaction was mainly affected by type of vegetation and year, but not by mulching treatment or their interactions (Table 5). Soil compaction was lower in 2019 than in 2020 (66.7 vs 81.8 kPa cm^-2^, with a soil moisture content of 30% and 23%, respectively). Furthermore, when averaged over the years, soil compaction was the highest for RV and the lowest for BS (Table 6). A significant effect of type of vegetation was also observed for tractor tyre print depth, but not for width (Table 5), with BS having higher values than all the other types of vegetation (Table 6).

**Table 6:** Effects of type of vegetation (M1 = grass-legume mixture 1, M2 = grass-legume mixture 2, S1 = *Schedonorus arundinaceus* blend, S2 = *Lolium perenne* blend, S3 = *Festuca rubra* blend, RV = resident vegetation) and bare soil (BS) on soil compaction, tractor tyre print depth, and total runoff sediments and P content of runoff sediments in 2019 and 2020.

Vegetation type	Soil compaction (kPa cm^-2^)	Tyre print depth (cm)	Total runoff sediments 2019 (g m^2^)	Total runoff sediments 2020 (g m^2^)	P content 2019 (mg kg^-1^)	P content 2020 (mg kg^-1^)
M1	69.86 b^1^	0.64 b	-^2^	-	-	-
M2	75.56 b	0.46 b	-	-	-	-
S1	70.08 b	0.64 b	-	7.12 c	-	1406.43 a
S2	73.61 b	0.52 b	6.12 b	17.21 b	949.87 b	1230.70 a
S3	79.19 b	0.46 b	-	-	-	-
RV	95.56 a	0.57 b	21.67 b	30.25 b	1279.87 a	1513.95 a
BS	54.72 c	1.03 a	259.33 a	616.12 a	826.41 b	845.20 b

^1^ Mean values with the same letters are not statistically different based on an LSD test at a probability level of 0.05.

^2^ Measurements were collected in reported plots only.

The total runoff sediments and their P contents were affected by type of vegetation cover (Table 5). BS had the highest total runoff sediment values in 2019 and 2020, while S1 had the lowest value in 2020 (Table 6). RV had the highest P content in 2019, BS the lowest in 2020.

**Fig 8:**
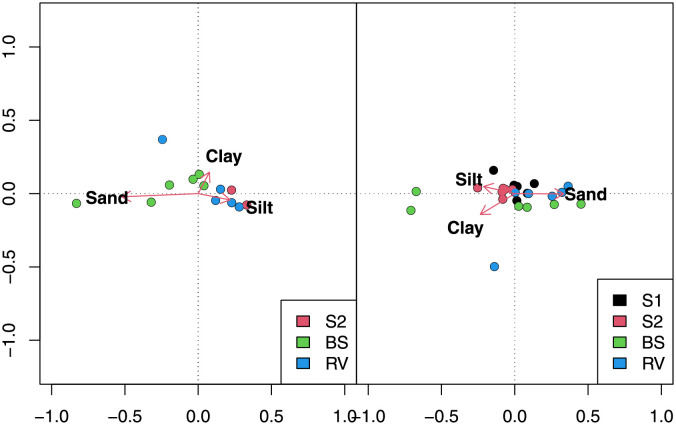
Principal component analysis of the particle size of sediments from runoff erosion in 2019 (on the left) and 2020 (on the right) with different vegetation types (S1 = *Schedonorus arundinaceus* blend, S2 = *Lolium perenne*, RV = resident vegetation) and bare soil (BS).

The type of vegetation also affected particle-size distribution of runoff sediments (Table 4). As can be seen in Fig 8, axis 1 separates the BS samples from S2 and RV, which was due to a lower amount of sand in the runoff sediments with S2 and RV (S2 Table). Fig 8 also shows a gradient of sand percentage moving along axis 1, and higher sediment values with BS and SV.

## Discussion

The observed effects depended mainly on the blends and mixtures used, and variations in botanical composition over time. Among the blends, *S*. *arundinaceus* was found to maintain a stable botanical composition throughout the study, whereas the other types of vegetation tested were rapidly invaded by other species. This result is similar to that of Miglécz et al. [79] who found that the composition of sown inter-rows changed from the first to the second year, with an increase in perennial and weed species in the second year. Similar observations for M1, M2, and S2 were mainly due to poor early-stage growth of *F*. *rubra* in M1 and M2, and *P*. *pratensis* in M2 during the winter immediately following sowing. As a result, only *L*. *perenne* was recorded in the plots sown with the mixtures in the surveys conducted in spring 2019, and the composition of these plots was similar to that of the plots sown with pure *L*. *perenne* (S2). *Lolium perenne* is known for its rapid establishment, especially compared with *F*. *rubra* and *P*. *pratensis* [80], and this may have given it the edge in competing with weeds during its establishment. *Festuca rubra* was recorded in the surveys conducted in the plots sown with pure *F*. *rubra* (S3), but its abundance throughout the study decreased from a mean relative frequency of 88% to 9% (S1 Table), confirming its low adaptability to the temperatures in the study area. In fact, this species is reported to have low heat tolerance, especially when combined with drought conditions [81–83]. Conspicuous changes were observed from spring to summer 2020 in the botanical composition of M1, M2, and S2, while there were changes in S3 only from spring to summer 2019. During hot summer months the sown species were gradually replaced by warm-season grasses, such as *Cynodon dactylon* (L.) Pers. and *Setaria viridis* (L.) Beauv. (S1 Table), which gained an advantage over the sown species from the decrease in green cover, as occurred in June 2019 (Fig 2) when temperatures were high, and precipitation was low (Table 1). The NDVI values (Fig 2) also highlight the suffering of all vegetation types in June 2019. However, the limited variability observed, especially in 2020, reflects a shift in botanical composition from sown cover to resident vegetation. *Schenodorus arundinaceus* was an interesting exception: the better overall performance (vegetation cover, environmental and pest resistance) and slower growth than all the other covers of this species are not similar to the results of other studies [47, 48], suggesting that mixtures give better ground cover persistence compared with monostands due to their greater resilience to biotic and abiotic stresses. However, our results substantiate the suggestion that choosing only one species with well-known, controlled characteristics simplifies agronomic management of inter-row vegetation [47], fertilizing and slashing in particular [14]. It is interesting that the relative frequency of *Trifolium repens*, which had a mean value of 47% in spring 2019 with M1 and M2, decreased in those mixtures and increased in S2 to reach the same values at the end of the study period (S1 Table). Furthermore, mulching did not affect any of the measured parameters, nor the botanical composition in the short-term, so not muclhing would save time and expenditure.

The influence of inter-row vegetation on the growth and yield of vines is still under discussion [84–89]. The results of our investigation suggests that vine parameters were not affected by the type of vegetation in the inter-row. Nor were any differences observed between grassed and bare soil inter-rows. Since the interaction between a woody and an herbaceous species might evolve over the time, long-term studies are needed to assess this [90]. In their review of studies on the effects of inter-row vegetation on several vineyard features, Vanden Heuvel and Centinari [90] mention only two that were conducted over the long term, both of which [86, 91] reported a gradual reduction in vine vegetative growth and an increase in pruning weight with grassed inter-rows compared with bare soil. However, these studies only covered a 6-7-year period, and inter-row vegetation has a greater negative effect on the vegetative growth of young vines than on old established vines due to the latter’s more developed root system [90].

Our results suggest that soil compaction was not directly affected by type of vegetation cover, but it was affected by tillage, whether in plots maintained with bare soil or when carried out to prepare the soil for sowing. Lack of vegetation led to greater soil compaction, while a dense, healthy sward reduced the effects of traffic. We found no differences in the depth of tractor tyre prints between plots tilled for sowing and untilled plots (i.e., covered by resident species).

In agreement with other studies [19–22, 31] we found reduced sediment runoff in grassed plots compared with tilled soil. Total runoff sediments seemed to be related to the percentage of vegetation cover. In both 2019 and 2020, RV and S2 exhibited the same values, which were lower than BS. However, in 2020, the green cover of S1 did not differ statistically from that of S2 (Fig 2), but the runoff sediments were much lower (Table 6). It is likely that vegetation cover provides a physical barrier that reduces runoff erosion [90]. Hence the importance of having a stable vegetation over time in terms of composition and coverage, as observed in plots seeded with *S*. *arundinaceus*. However, numerous studies have reported that the positive effects of permanent swards only become apparent after several years, the time needed to maximise soil organic matter accumulation [11, 92] or become stable [93, 94].

## Conclusions

The study revealed other species rapidly invading the tested vegetation types, with conspicuous changes in botanical composition in summer when high temperatures and low precipitation favoured C4 annual grasses. Only *S*. *arundinaceus* was able to successfully compete with these species, maintaining the initial botanical composition over the entire study period. Because of its high persistence, *S*. *arundinaceus* gave high protection against soil erosion, and had the lowest growth rate thus requiring fewer cuttings. Our results also show that mulching has no effect on the inter-row vegetation or even on the vineyards. Similarly, vine parameters were not affected by the type of vegetation in the inter-row, although long-term studies are needed to evaluate the evolution of the interaction between a woody and a herbaceous species. Soil compaction and soil erosion were not directly affected by the type of vegetation cover but were affected by tillage. Absence of vegetation led to greater soil compaction and erosion. The conclusion we draw from these findings is that species selection plays a crucial role in inter-row vegetation management and in minimising environmental impacts. The high adaptability of *S*. *arundinaceus* to the local conditions makes this species the most suitable of all the vegetation types tested for inter-row cover.

## Supporting information

S1 Table Relative abundances of plant species (averaged over 3 replicates) recorded in the botanical surveys conducted in June 2019, August 2019, June 2020 and August 2020. M1 = grass-legume mixture 1, M2 = grass-legume mixture 2, S1 = *Schedonorus arundinaceus* blend, S2 = *Lolium perenne* blend, S3 = *Festuca rubra* blend, RV = resident vegetation, BS = bare soil.
(XLSX)

S2 Table Particle-size distribution (averaged over 3 replicates) determined in soil erosion samples collected in 2019 and 2020. S1 = *Schedonorus arundinaceus* blend, S2 = *Lolium perenne* blend, RV = resident vegetation, BS = bare soil.
(XLSX)
